# Application of Super Absorbent Polymer and Plant Mucilage Improved Essential Oil Quantity and Quality of *Ocimum basilicum* var. Keshkeni Luvelou

**DOI:** 10.3390/molecules25112503

**Published:** 2020-05-28

**Authors:** Somaye Beigi, Majid Azizi, Marcello Iriti

**Affiliations:** 1 Department of Horticultural Science, Faculty of Agriculture, Ferdowsi University of Mashhad, Mashhad 9177948974, Iran; sbeigi61@gmail.com; 2 Department of Agricultural and Environmental Sciences, Milan State University, via G. Celoria 2, 20133 Milan, Italy

**Keywords:** basil, essential oil content, hydrophilic polymer, volatile oil quality, water use efficiency

## Abstract

One of the major factors limiting the production of medicinal plants in arid and semi-arid areas is water deficit or drought stress. One-third of the land in the world is arid and semi-arid and is inhabited by nearly 4 × 10^8^ people. *Ocimum basilicum* (sweet basil) is a valuable medicinal plant that is sensitive to water deficit, and water shortage negatively affects sweet basil yield and quality. Water availability in the root zone of basil could ameliorate the negative effects of water shortage. To the best of our knowledge, although the effects of hydrophilic polymers (HPs) have been studied in different agricultural crops, the effects of HP application in medicinal plants have not been previously investigated. This investigation was conducted to explore the effects on water use efficiency when using Stockosorb^®^ (STS) and psyllium seed mucilage (PSM) as hydrophilic polymers (HPs) and the effects of these HPs on essential oil quality, quantity, and yield. The research was set up in a factorial experiment on the basis of completely randomized block design with three replications. We used two HPs, STS (industrial) and PSM (herbal), with two methods of application (mixed with soil, mixed with soil + root) at four concentrations (0%, 0.1%, 0.2%, and 0.3% (*w*/*w*)). Results showed that the STS and PSM significantly increased the dry herb yield (both shoot and root) in comparison to the control, and the improving effect was higher when these HPs were mixed with soil + root. The highest dry herb yield (6.74 and 3.68 g/plant for shoot and root, respectively) was detected in the PSM at 0.1% mixed with soil + root. There was not any significant difference in dry herb yield among PSM (0.1%), PSM (0.2%), and STS (0.2%) when mixed with soil + root. Soil application of PSM and soil + root application of STS at a concentration of 0.3% increased the Essential Oil (EO) content almost three-fold in comparison to the control (0.5% and 0.52% to 0.18% *v*/*w*, respectively). The maximum essential oil yield was recorded in plants treated with STS (0.2% in) or PSM (0.1%) by soil + root application (0.21 and 0.19 mL/plant, respectively). PSM at concentrations of 0.1% and 0.2% (mixed with soil + root) showed the highest water use efficiency (1.91 and 1.82 g dry weight (DW)/L H_2_O, respectively). STS mixed with soil also significantly improved water use efficiency (WUE) in comparison to the control. The application of these HPs improved the quality of sweet basil essential oil by increasing the linalool and decreasing the eugenol, epi-α-cadinol, and trans-α-bergamotene content.

## 1. Introduction

Medicinal and aromatic plants are of prime economic importance because of the continuous and increasing demand for their products by local and foreign markets [1]. Basil (*Ocimum basilicum* L.), part of the Lamiaceae family, is one of the most important medicinal plants [2] that can be used for dry leaves and flowers, its essential oil, fresh vegetables, and as an ornamental plant [3]. Its essential oil is extensively employed to add a distinctive aroma and flavor to food and can be used in cosmetics or pharmaceuticals for an enormous number of ailments, including convulsion and diarrhea [4,5]. Additionally, previous studies reveal antiviral, antimicrobial, antioxidant, and anticancer properties (4). Basil is cultivated all over the world because it is an underutilized crop with many different uses and has great potential for use as an alternative horticultural crop [6]. The export of sweet basil dry leaves, essential oils, and chemical derivatives annually is worth 10,000 tons [7,8,9]. Basil is very sensitive to water stress [10] and displays wilt symptoms shortly after water deficiency because it has large leaves and a high water consumption that can be more than 800 mm [11]. The main constituents of basil essential oil are methyl chavicol, eugenol, and linalool [12,13]. The amount of each of these chemical constituents varies depending on the species, variety, and chemotype [7,8,9,14,15].

In many regions of the world, including Iran, drought stress is one of the largest factors that decreases agricultural crop production more than any other single environmental factor [16]. Therefore, the water holding capacity of soil and water use efficiency are important issues for increasing yield, especially in arid and semi-arid regions [17]. Nowadays, the application of water-holding compounds like superabsorbent polymers or hydrophilic polymers (HPs) in agricultural and horticultural fields is a simple method to remedy these issues [18]. Superabsorbent polymers can be divided into two classes, on the basis of original source, natural and synthetic. Natural types are polysaccharide-based or polypeptide-based (gelatin and collagen). Synthetic types are petrochemical-based [19]. Hydrogels are obtained by chemical stabilization of HPs (polyacrylamide) in a tridimensional network [20,21]. They can retain a large amount of water (400–1500 g of water/1 g dry hydrogel) and nutrients [22,23]. Superabsorbent polymers can reduce irrigation frequency up to 50% [23] and reduce water stress in plants, improving plant growth characteristics [24,25]. Their use has also been shown to increase the effects of fertilizers and pesticides [26].

Hydrophilic polymers (HPs) are either natural or synthetic. Some natural HPs are polysaccharides, humus, polyuronids, and alginic acid. Mucilages are herbal polysaccharides and commonly include carbohydrates such as arabinose, xylose, and uronic acid along with cellulose and other water-soluble polysaccharides [27]. Mucilage has a high water-holding capacity and improves the mechanical contact between soil and roots, avoiding the formation of gaps as roots shrink in response to high transpiration or drought stress [28]. Positive effects of using superabsorbent polymers have been recorded for the production of different plants such as *Pinus halepensis* seedlings [22], *Agrostis tenuis* [29], *Linum usitatissimum*, and *Matricaria chamomilla* [30]. Waste pulp from the Cassava plant, with water absorption almost 1000 times their dry weight, can be used in soils as superabsorbent polymers according to Masud et al. (2013) [31]. Research has also shown that the application of superabsorbent polymers caused an increase in unsaturated fatty acids such as linoleic acid and reduced saturated fatty acids in six cultivars of canola, ultimately leading to an increased quality of canola oil [32]. Most studies focused on the effect of HPs only on plant growth parameters and the effects of HPs on medicinal plants, and their production of active ingredients has not been extensively investigated. Therefore, more research is needed on medicinal plants for their reaction to HPs, especially for production in arid areas. The aim of medicinal plant production is phytochemical extraction and, in this regard, increasing the yield (dry herb yield), yield components, and phytochemical content is very important. Therefore, the aims of this investigation were to study the effects of HPs on water use efficiency, dry matter, leaf area, essential oil quantity, quality, and yield of basil, and to evaluate its potential application for cultivation of the plant in arid regions.

## 2. Results and Discussion

### 2.1. Total Dry Matter (Biomass)

**Figure 1:**
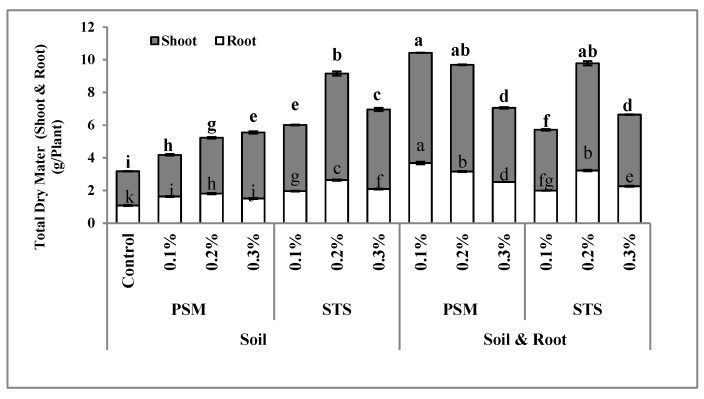
The interaction effect of hydrophilic material, application methods, and concentrations on total dry matter of sweet basil. Bars with different letters on top (shoot or root) show the significant difference by Duncan’s multiple range test at *p* ≤ 0.05; STS: Stockosorb; PSM: Psyllium seed mucilage.

The effect of the treatments on total dry matter (biomass) was statistically significant (*p* ≤ 0.01). According to the results (Figure 1), the lowest biomass (3.18 g/plant) was observed in the control plot and the highest biomass (10.42 g/plant) belonged to plants treated with psyllium seed mucilage (PSM) at a concentration of 0.1% (soil + root application). In Stockosorb^®^ treatments, regardless of the method of application, the medium concentration (0.2%) produced higher biomass. In PSM treatments, increasing the concentration from 0.1% to 0.3% by soil application improved the biomass; however, in soil + root application, we observed a reversed trend with the highest biomass detected in lower concentrations of PSM, and there were no significant differences between 0.1% and 0.2% concentrations. There was no significant difference between biomass produced in the treatment of Stockosorb^®^ mixed with soil + root and biomass produced in the plot treated with PSM at the concentration of 0.2% (soil + root application).

Improving the physical and chemical properties of soil by the application of hydrogels was confirmed, as well as the increased water use potential during the establishment of crops [33,34]. Improving the water holding capacity increased plant performance and yield in other agricultural crops such as millet [35] and groundnut [36]. The buffering environment and water conservation in soil upon application of hydrogel are the main reasons for increasing the plant dry weight after using these types of superabsorbent polymers [37]. It has also been claimed that hydrogel reduced the fertilizer leaching through interaction between the hydrogel and nutrient of fertilizers [38]. In the present study, nutrient absorption was not evaluated, but the increase in the dry matter upon application of the superabsorbent indirectly showed the improved nutrient absorption. The increased yield of biomass after application of superabsorbent polymers has also been previously reported [24,25]. In any case, the application of the hydro-absorbent in agriculture and horticulture needs economic evaluation [37]. Application of hydrogel on the root (when applied as root dip only) coats the fine roots and protects the root against desiccation, especially after seedling transplanting. One explanation is that the hydrogel could act as natural root exudates and polymeric mucilage produced by healthy roots [39]. Other researchers confirmed that root dip application of hydrogels weakens the water potential drop at the root–soil interface and decreases the energy needed to absorb water from soil [28]. The method of the superabsorbent polymer also improves the root-to-soil contact and filling-in air space around seedling roots after transplanting [39]. The optimum concentration is very important for obtaining fast results and decreasing the amount of hydro-absorbent needs for field unit area. In total, using the PSM mixed with soil + root improved dry matter more than using it only in soil, but for Stockosorb^®^ (STS), it is better to use in soil. For cultural practices in horticulture, it will be easy to add synthetic HPs during soil preparation before plant cultivation when direct seeding is proposed, whereas, for indirect cultivation (seedling transplanting), soil + root application will be recommended. The method of application in soil depends on the cultivation system. In furrow cultivation, banding of the polymer needs only 46 kg ha^−1^. For fruit trees, several injection machines have been designed to apply dry polymers or hydrated polymers in the root zone [40]. In greenhouse production of horticultural crops, nursery crops, seedling production, or rooted cutting, the method of application is adding to soil, soil + root, or soilless media [40].

### 2.2. Water Use Efficiency (WUE)

Water use efficiency is expressed as the amount of each crop yield (kg) that could be produced from the unit volume of water (cubic meter or liter) [29]. It depends on soil water holding capacity, irrigation efficiency, and plant transpiration behavior. Increasing the WUE in arid and semi-arid regions is very important because of water limitation.

**Figure 2:**
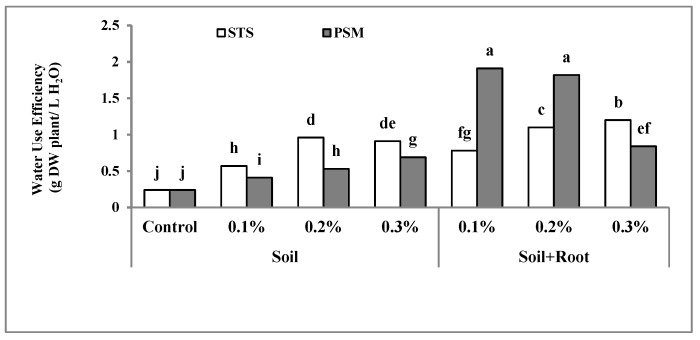
The interaction effect of hydrophilic material, application methods, and concentration on water use efficiency. Bars with different letters on top show the significant difference by Duncan’s multiple range test at *p* ≤ 0.05. STS: Stockosorb; PSM: Psyllium seed mucilage.

According to the results of analysis of variance, in comparison to the control, all treatments increased water use efficiency (WUE) significantly (*p* ≤ 0.01). For the soil application method, increasing the concentration of both PSM and Stockosorb^®^ from zero (control) to 0.3% improved WUE (from 0.24 to 0.96 g DW/L H_2_O for STS and from 0.24 to 0.69 for PSM). We found Stockosorb^®^ to be significantly superior to PSM in regard to WUE (Figure 2). The WUE in soil + root application was higher than that of soil application in both PSM and Stockosorb^®^, and the PSM was superior to Stockosorb^®^ except at the highest concentration (0.3%) where STS was better than PSM. The highest WUE (1.91 g/L) was observed in plants treated with PSM at a concentration of 0.1% (mixed with soil + root); furthermore, there was no significant difference with PSM at a concentration of 0.2% (mixed with soil + root) (Figure 2). The lowest WUE (0.24 g/L) resulted from the control plot. The application method of Stockosorb^®^ had no significant effect on WUE, but in PSM, the method of application had a significant effect on the WUE with soil + root application, resulting in superior performance. 

Hydrogel application by preventing water leaching from soil, decreasing evaporation and infiltration, improved the WUE [34]. Increasing the level of hydrogel increased the WUE in pearl millet [35] and *Pinus halepensis* [22]. In citrus fruit, application of Stockosorb increased the period of water availability for 15 days, and this was in accordance to soil water holding from 28.74% to 34.63% [41]. Greenhouse crop producers also search for methods that could reduce the water requirement and also decrease the watering time during each production season. They use hydrogels or water-absorbent polymers in soil or soilless media such as holly, juniper, and azalea [42]. Several authors confirmed an increase in irrigation efficiency through the application of superabsorbent polymers [20,26,29]. The application of PSM in soil + root in comparison to the application in soil alone was more efficient for increasing the WUE. This is probably because of better water retention in the root zone.

### 2.3. Leaf Area

**Figure 3:**
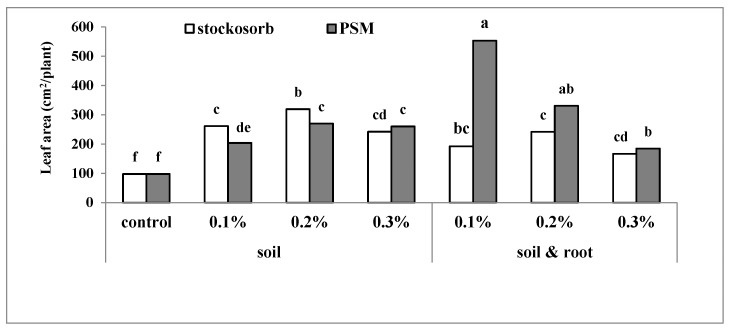
The interaction effect of hydrophilic material, application methods, and concentration on leaf area of sweet basil. Bars with different letters on top show the significant difference by Duncan’s multiple range test at *p* ≤ 0.05; STS: Stockosorb; PSM: Psyllium seed mucilage.

According to the result, the leaf area/plant was affected significantly by all treatments in comparison to the control treatment (*p* ≤ 0.01). In the soil application method, both hydrophilic materials increased the leaf area significantly, and at low (0.1%) and medium (0.2%) concentration, Stockosorb^®^ was more efficient than PSM except at the highest concentration where there were no significant differences between Stockosorb^®^ and PSM. In the soil + root application method, increasing the PSM concentration decreased the leaf area from 453.1 (in 0.1%) to 184.6 cm^2^/plant (in 0.3%). With low and medium concentrations of PSM, the leaf area was significantly higher than Stockosorb^®^-treated plants. The highest and the lowest leaf area (553.13 and 98.02 cm^2^/plant) were produced in plants treated with PSM (0.1% mixed with soil + root) and the control plot, respectively (Figure 3). Increasing leaf area after superabsorbent polymers application was also reported in *Acacia victoriae* [25], soybean [24], and *Pinus halepensis* [22]. Decreasing leaf area upon increasing the PSM concentration (in soil + root application method) may be related to the negative effect of this high concentration of PSM (0.2% and 0.3% PSM) for direct application on root, and it may be higher than the optimum concentration or more than the tolerant level of the basil root. Other possible explanations for getting this effect may be related to the induction of a high matric water potential effect around plant roots that belongs to the nature of the PSM. To the best of our knowledge, there is no report on the use of PSM on the soil + root of basil or other plants for comparison, and it may related to the concentration that needs more research for evaluating the lower concentration of PSM when applied on soil + root of basil. The soil + root application of hydrogel could be useful in the indirect cultivation of horticultural crops (seedling transplanting). By comparison to other evaluated factors, the 0.1% PSM is a good treatment, and more research needs to evaluate lower PSM concentrations. On the other hand, in the present work, nutrient uptake in different treatments was not evaluated, and much work remains to be done for determining the mode of action of the superabsorbent polymer on the leaf area 

### 2.4. Essential Oil Content (%v/w)

**Figure 4:**
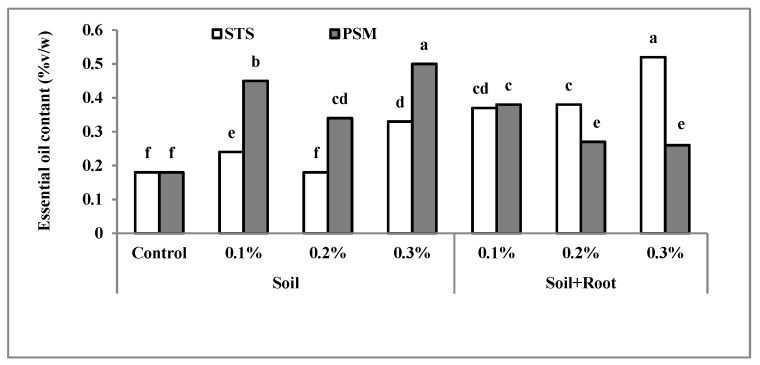
The interaction effect of hydrophilic material, application methods, and concentration on essential oil content (%*v*/*w*). Bars with different letters on top show the significant difference by Duncan’s multiple range test at *p* ≤ 0.05; STS: Stockosorb; PSM: Psyllium seed mucilage.

The results of analysis of variance indicated that essential oil content was significantly affected (*p* ≤ 0.01) by all treatments. The highest essential oil content was produced in plants treated with PSM and Stockosorb^®^ at a concentration of 0.3%, and the lowest essential oil content (0.18% *v*/*w*) was extracted from control plants (Figure 4). According to the results in both application methods, increasing the concentration of Stockosorb^®^ increased essential oil content. The essential oil content of plants treated with PSM increased by increasing the PSM concentration mixed with soil, while it decreased when it mixed with soil + root (Figure 4). Essential oil biosynthesis in herb was controlled by two main factors, endogenous and exogenous. The former related to the plant genetic characteristic but the latter related to environmental conditions such as light, temperature, nutrient level, and moisture. The exogenous factors may exert their effects directly or indirectly [43]. 

According to the Council of Europe (2001) [44], the standard essential oil content of basil on the basis of dry weight should be 0.8% *v*/*w* [45]. Overall, the yield and essential oil percentage was severely reduced by the water stress conditions. Medium water stress has been shown to increase secondary metabolites without negative effects on physiological activities, growth, and flowering [2,46]. Increased essential oil content in *Lippia citriodora* through the application of balanced fertilization + biofertilizer + superabsorbent polymer as a water preservative in soil and mild stress has also been reported [47]. Mild water stress in arid and semi-arid regions demonstrated increased activity of phenylalanine ammonia lyase (PAL) and this protein synthesis shift toward phenol and final shift to essential oil. 

### 2.5. Essential Oil Yield 

**Figure 5:**
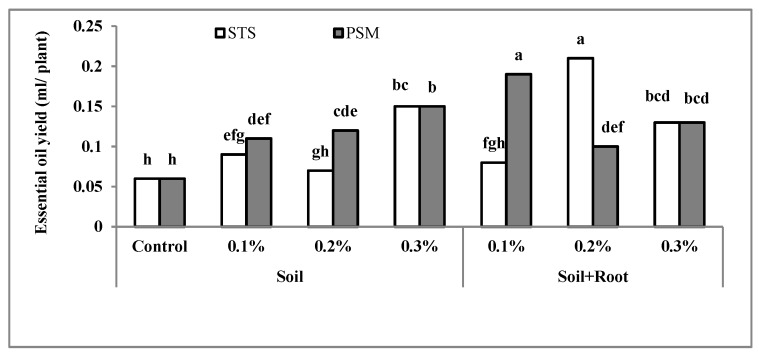
The interaction effect of hydrophilic material, application methods, and concentration on essential oil yield. Bars with different letters on top show the significant difference by Duncan’s multiple range test at *p* ≤ 0.05; STS: Stockosorb; PSM: Psyllium seed mucilage.

According to the results, significant effects (*p* ≤ 0.01) were recorded in the case of essential oil yield by all treatments. According to Figure 5, the lowest essential oil yield (0.06 mL per plant) was observed in control plants and the highest (0.21 mL per plant) belonged to plants treated with Stockosorb^®^ at 0.2% (soil + root application); furthermore, there was no significant difference with PSM at a concentration of 0.3% (soil + root application). All treatments in comparison to the control decreased the adverse effect of water shortage of the root zone and kept soil water content near to the field capacity. Treatments provided greater support of the plants’ water requirements for proper growth and development, increasing essential oil yield in comparison to the control when the Stockosorb^®^ and PSM applications were at a concentration of 0.2% and 0.3% (mixed with soil + root), respectively. Essential oil yield is directly related to the essential oil content (on the basis of g/100 g DW) and herb yield. In turn, herb yield depends on cultivar, harvest season, and soil water availability [30,45]. Essential oil yield in published articles on medicinal plants has been presented as kg/ha [48] or mL/plant. The former depends on the number of plant/ha and is related to the plant density that is affected by geographical conditions, especially light intensity, whereas the latter could be presented in pot experiments or small plot research projects. In our study, the essential oil yields in different treatments were presented as mL/plant in the range of 0.06–0.21 mL/plant. Ichimura et al. (1995) [49] by studying the effect of P fertilizer on the growth and essential oil yield of basil showed that phosphorus fertilizer could increase the essential oil yield from 0.006 to 0.04 mL/plant in spring and from 0.035 to 0.06 mL/plant in summer. A higher level of essential oil yield in our study could be related to the cultivar and climatic conditions. They did not express the basil cultivar in their research. Zheljazkov et al. (2008) [48] studied 38 accessions of basil and reported an essential oil yield in the range of 3.1–58.8 kg/ha. The plant density in their study was near 3 plant/m^2^. Other researchers have verified increased essential oil yield by increasing water availability in soil [6]. Shii et al. [50] showed that Stokosorb treatment prolonged the water supply to plant duration and increased growth in *Populus popularis*. In our study, growth parameters of basil improved after the use of water preservative compounds in soil and indirectly increased essential oil yield by increasing herb yield (Figure 1). 

### 2.6. Essential Oil Quality

Results of the GC-MS analysis of the basil essential oil are shown in Table 1 and Table 2. In total, 49 to 52 components were determined in the essential oil of basil treated with Stockosorb^®^ mixed with soil + root that represented 98% of the essential oils. The major constituents of the essential oil in the samples were linalool (from 53.4% to 63.4%), 1,8-cineole (from 3.7% to 7.8%), epi-α-cadinol (from 5.1% to 5.9%), trans-α-bergamotene (from 2.7 to 3.1%), methyl chavicol or estragole (from 1.2% to 3.9%), and eugenol (from 2.1% to 2.5%) (Table 1). In addition, 37 to 45 compounds were identified in the essential oil of *O. basilicum* treated with Stockosorb^®^ mixed only with soil (98.5% of the total essential oils). The main constituents found in the essential oil of these samples were linalool (from 52.4% to 55.2%), 1,8-cineole (from 3.3% to 5.1%), epi-α-cadinol (from 4.98% to 6.6%), trans-α-bergamotene (from 3.5% to 5.3%), methyl chavicol (from 1.9% to 2.9%), and eugenol (from 2.8% to 4.9%) (Table 2). In *O. basilicum* treated with PSM mixed with soil + root, 35 to 46 constituents were determined (99% of the total essential oils), linalool (from 57.4% to 63.6%), 1,8-cineole (from 4.8% to 8.8%), epi-α-cadinol (from 4.2% to 5.3%), trans-α-bergamotene (from 3.5% to 5.3%), methyl chavicol (from 1.0% to 4.2%), and eugenol (from 2.3% to 3.1%), which are reported as the main components of volatile oils (Table 1). While in plants treated with PSM mixed only with soil (at 0.1%, 0.2%, and 0.3% concentration), 41 to 50 constituents were observed (99% of the total essential oils) with the dominant components consisting of linalool (from 59.9% to 62.8%), 1,8-cineole (from 3.6% to 5.1%), epi-α-cadinol (from 3.8% to 6.2%), trans-α-bergamotene (from 2.1% to 2.4%), methyl chavicol (from 0.2% to 9.4%), and eugenol (from 1.3% to 3.6%) (Table 2). 

**Figure 6:**
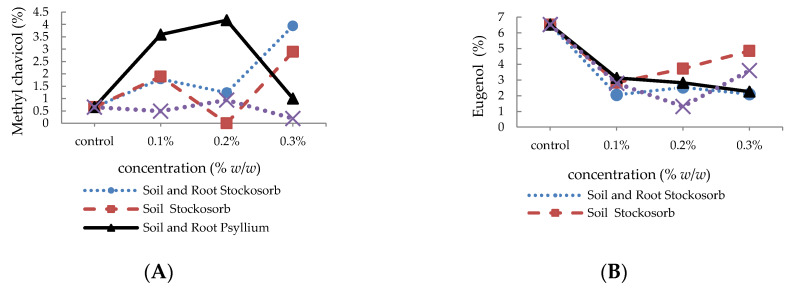

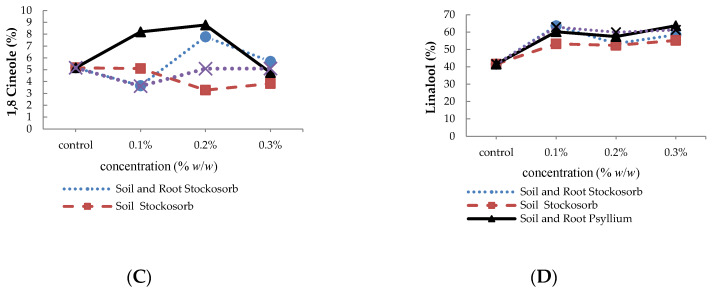
Changes in content of methyl chavicol (**A**), eugenol (**B**), 1,8 cineole (**C**), and linalool (**D**) in basil, after treatment with HPs.

According to the GC-MS analysis of the essential oils (Figure 6), regardless of the method of application of HPs, linalool and eugenol (Figure 6D,B) contents of basil essential oils increased and decreased, respectively. The methyl chavicol content of plants treated with PSM (in soil application methods) was stable (Figure 6A), but application of Stockosorb^®^ (in both methods) increased the constituent drastically. Plants treated with PSM (in both methods and in different concentrations) had a greater amount of major constituents of essential oil than Stockosorb^®^-treated plants. The highest amount of linalool (Figure 6D) and methyl chavicol (Figure 6A) was obtained in plants treated with PSM mixed only with soil at 0.1% and 0.2% concentrations, respectively. Plants treated with PSM mixed with soil + root (at 0.2% concentration) had the maximum 1,8-cineole (Figure 6C). Timmerman et al. (1984) [51] expressed, on the basis of more than 200 essential oil analysis reports, the maximum level of linalool synthesized in wet growing conditions in comparison to dry growing conditions. Bettaieb et al. (2009) [52] reported that the highest amount of 1,8 cineole was detected in mild drought stress, which was consistent with the results of this study. Therefore, increased 1,8 cineole and linalool constituents were due to applying the superabsorbent polymer and increasing water availability in the soil and root zone.

*Ocimum basilicum* essential oils are mainly originated from mevalonic acid or phenylpropanoid pathways. The biosynthesis of volatile oil constituents was catalyzed by different enzymes (Scheme S1 in Supplementary Materials).

Lawrence (1988) [53] classified four major essential oil chemotypes of basil: (1) methyl chavicol-rich, (2) linalool-rich, (3) methyl eugenol-rich, (4) methyl cinnamate-rich, and also numerous subtypes. Bruneton (1993) [54] obtained results that were generally different to the literature findings concerning the major compounds. The observed differences might be the result of different growing environments, genetic factors, or chemotypes, as well as other potential factors that can influence the oil composition. Lee et al. (2005) [55] reported that, regardless of environmental, genetic, or chemotype factors, 1,8-cineole, methyl cinnamate, methyl chavicol, and linalool are generally the main compounds responsible for the typical basil aroma [53]. The chemical composition of various cultivars of sweet basil essential oils was evaluated by Kartnig and Simon (1986) [56] in three harvest times. They found that in all varieties and all harvest times, linalool was the main constituents (in the range of 39.8–75.5%). Simon et al. (1992) [57] evaluated the essential oil content and composition of sweet basil upon water stress treatments and confirmed that water stress affects the chemical composition of basil essential oil. They showed that the relative percentage of linalool and eugenol decreased in moderate stress in comparison to the control (from 29.5% to 16.3% and 4.3% to 0.1%, respectively) but methyl chavicol instead increased (from 30.3% to 49.9%). They finally concluded that even mild water stress can increase essential oil content and change the essential oil constituents. In the present research, by increasing the HP concentration (decreasing matric potential of water), the linalool content increased but eugenol decreased and the methyl chavicol content did not show a defined trend. The results may be related to the method of representing each constituent (relative percentage) that increases in one or two constituents should force the other constituents to decrease. For a deeper explanation of the effect of HP application on essential oil constituents, the evaluation of enzymes responsible for the biosynthesis of each compound, and also independently measuring each compound content (not relative content), is necessary.

There are consumption restrictions for some compounds of basil essential oil such as methyl chavicol (or estragole), eugenol (phenolic compounds), eucalyptol, and some alkyl benzene compounds like methyl eugenol and, notably, safrole (a phenylpropanoid ether) (Table S1 in Supplementary Materials). According to the information of Gubta et al., sweet basil essential oil, with a low content of methyl chavicol (<7%) and high linalool content, has been recommended [58]. Plants rich in linalool (a terpene alcohol) demonstrate potent effects on the central nervous system in vivo, including sedative, spasmolytic, and hypothermic activity. Linalool also has a local anesthetic effect [54]. The greater antioxidant potential of *O. basilicum* essential oil might be correlated to the high content of linalool in this oil [45]. According to the results in this study (Table 1 and Table 2), the linalool constituent was enhanced by the use of both polymers (PSM and Stockosorb^®^).

## 3. Materials and Methods 

### 3.1. Experimental Design

This research was conducted as a pot experiment at the Department of Horticultural Science‚ College of Agriculture‚ Ferdowsi University of Mashhad‚ Mashhad‚ Iran, (59°38′ E and 36°16′ N; elevation 989 m; mean annual rainfall 255.2 mm). Long-term averages of maximum and minimum temperature were 22 °C and 8.9 °C‚ respectively. The soil used in the experiment was analyzed at the Department of Soil Science of Ferdowsi University of Mashhad on the basis of the standard method [59]. The results are presented in Table 3. The research was arranged in a factorial experiment with 16 treatments on the basis of the randomized complete block design (RCBD) with three replications. The treatments were: Use of two HPs (Stockosorb^®^ and psyllium seed mucilage, “PSM”) with two methods of application (mixed with soil or mixed with soil + root) at four concentrations (0, 0.1%, 0.2%, and 0.3% *w*/*w*). Data were subjected to analysis of variance (ANOVA) followed by Duncan’s multiple range test at *p* ≤ 0.05 using SAS software v.9.4 (SAS institute, North Carolina State University, North Carolina, NC, USA).

### 3.2. Experimental Procedure 

Seeds of *O. basilicum* var. “Keshkeni luvelou” were sown in a field on 10 June for production of seedlings (15 cm). Stockosorb^®^ was purchased from the local agricultural supplement market of Mashhad. The superabsorbent polymer could be used in the two methods, mixing with soil or root dipping [39]. For soil application, the definite amount (0, 7, 14, and 21 g/pot) of Stockosorb^®^ was added to the soil and mixed completely. For soil + root application, after preparing the soil as above, the seedlings were dipped (for 2 min) in different respective concentration solutions of Stockosorb^®^ before transplanting [39]. 

For soil application of PSM, 7, 14, and 21 g dry seeds of *Psyllium ovata* were ground and mixed with the soil completely. For the soil + root application of PSM, after preparing the soil as above, the seedlings were dipped (for 2 min) in the different respective concentration solutions of PSM before transplanting. PSM solutions were prepared on the basis of the Marlett and Fischer (2005) method by some modification [60]. Briefly, different weights of grounded *P. ovata* seeds (0.1, 0.2, and 0.3 g) were dissolved in 100 mL distilled water for 10 h at ambient temperature (20 ± 1 °C). Then, the solutions were centrifuged at 10,000 g for 30 min. Next, the seedlings were dipped in the solutions for 2 min and transferred to the pot (30 cm diameter and 10 kg capacity) for growing [39]. Plant were irrigated every two days at a rate of 1000 mL/pot until flowering occurred [13]. The amount of water used for each treatment is presented in Table 4. The goal was to determine the best water use efficiency in the stress of the plants, according to the treatments. During the flowering periods, different parameters were evaluated as follows. 

### 3.3. Dry Matter and Leaf Area

During the full flowering period, all plants were cut at 3 cm above the soil level and weighed. Next, the leaf area of each plant was measured using a leaf area meter (Delta T). All samples were then dried at room temperature (25 ± 2 °C). 

### 3.4. Water Use Efficiency

The water use efficiency was calculated according to the dry herb biomass (g) in each plot and water amount for their irrigation until the flowering stage on the basis of the following equation [61]:

(1) $$\mathrm{Water}\;\mathrm{Use}\;\mathrm{Efficiency}\;(g/L)=\frac{\mathrm{Dry}\;\mathrm{matter}\;\left(g\;\mathrm{DW}\;\mathrm{plant}\right)}{\mathrm{Amount}\;\mathrm{of}\;\mathrm{water}\;\mathrm{used}\;\left(l\right)}$$

### 3.5. Essential Oil Extraction

The aerial plant parts were harvested during the full flowering stage and shade-dried at room temperature (25 ± 2 °C). Then, 30 g of dry biomass was used for the extraction of essential oils for each treatment replication. The essential oils were extracted by hydro-distillation using a Clevenger apparatus and presented by %*v*/*w* [62]. The essential oil yields were calculated by the volumetric method (essential oil percentage × dry matter of plant). 

### 3.6. Identification of Essential Oil Constituents

Essential oil constituents of the samples were determined by GC and GC/MS analysis according to the previous published paper with some modification [63].

GC analysis was carried out using an Agilent-Technologies−7890A gas chromatograph (Santa Clara, CA, USA) equipped with a HP-5 column (30 m × 0.32 mm i.d) film thickness (0.25 μm). The oven temperature started from 60 °C and was then programmed to rise to 210 °C at a rate of 3 °C /min, and then 210 °C to 240 °C at 20 °C/min and held for 8.5 min. Injector and detector (FID) temperatures were 280 °C and 290 °C, respectively; N_2_ was used as a carrier gas with a linear velocity of 1 mL/min. The split ratio was 1:50.

GC/MS analyses were carried out on a GC/MS (Agilent Technologies-5975C-MS, 7890A-GC, Santa Clara, CA, USA) equipped with HP-5MS (30 m × 0.25 mm (i.d) × film thickness 0.25 μm capillary column). The oven temperature was programmed as follows: From 60 °C to 210 °C with a rate of 3 °C/min, then increased to 240 °C with a rate of 20 °C/min, and then the final temperature kept for 8.5 min, run time: 60 min. The electron ionization energy was 70 eV in the electronic ionization (EI) mode, ion-source: 230 °C, detector: MS, interface line temperature: 280 °C, injector: 280 °C, split ratio: 1:50, carrier gas: He, 1 mL/min, mass range: 50–480 amu.

The percentages of essential oil components were calculated by the area normalization method. The components of the oil were identified by comparison of retention indices (RRI, HP-5) in their mass spectra with those of an Adams library and sorted in NIST and Wiley libraries or with authentic compounds reported in the literature. Retention indices were determined using retention times of *n*-alkanes that were injected after the essential oil under the same chromatographic conditions. Finally, all data except essential oil constituents were subjected to analysis of variance (ANOVA) using JMP8 software and LSD tests at 5% levels for mean comparison.

## 4. Conclusions

All treatments showed significant effects on basil performance, quantity, and quality of its essential oil and also on water use efficiency over the control treatment. Both polymers (regardless of concentration and method of application) exhibited favorable effects on the chemical composition of essential oil, especially increasing linalool and decreasing eugenol compared to the control. These effects would suggest a different modulation of the secondary metabolic pathways involved in biosynthesis of the essential oil constituents, by stimulating the mevalonate route (leading to linalool) and suppressing the phenylpropanoid pathway (leading to eugenol). The level of methyl chavicol and 1,8-cineole after the application of these polymers did not show a definite trend, but the content was in the allowable limit. PSM application (in both methods and in different concentrations) increased the amount of major constituents more than Stockosorb^®^ application. This is a promising aspect because PSM is a natural product. Results of this study showed that the major constituent in the essential oil of treated plants by both polymers is linalool. In addition, compounds with restrictions because of toxicity, such as camphor, methyl eugenol, 1,8-cineole, and methyl chavicol, were in the allowable limit, while in the control treatment, eugenol was not within the allowable limit [58] (Table S2 in Supplementary Materials). Although PSM treatment (mixed with soil + root) had the best results for the vegetative traits and water use efficiency, the highest amount of methyl chavicol and linalool was found in plants treated with PSM. The quantity and essential oil yield were highest in plants treated with Stockosorb^®^ mixed with soil + root. However, selection of the polymers (Stockosorb^®^ and PSM) by the farmer depends on the application method and also depends on the cultivation system as direct seeding or seedling transplanting. Degradability of the polymers in soil (shelf life), the HP price, and the availability of the polymer in the market are very important issues to select each HP by farmers. In essence, these results showed that natural compounds could be a good alternative to chemical compounds for increasing water availability in the soil and increasing water use efficiency, especially in arid and semi-arid agricultural environments. In these terms, cultivation of medicinal and aromatic plants could be improved in countries with arid and semi-arid conditions, with economic benefits for farmers in the face of low production costs.

## Figures and Tables

**Table 1:** Essential oil components of treated basil by two industrial and plant hydrophilic polymers (HPs) in various concentrations with application method in soil and root.

No.	Essential Oil Components	RI *	Control	Stockosorb^®^			PSM		
				0.1%	0.2%	0.3%	0.1%	0.2%	0.3%
1	α-Thujene	929	-	-	0.01	-	-	-	-
2	α-Pinene	933	0.21	0.10	0.41	0.21	0.16	0.40	0.07
3	Camphene	948	0.07	0.05	0.12	0.07	0.03	0.09	-
4	Sabinene	972	0.27	0.12	0.41	0.25	0.25	0.43	0.09
5	β-Pinene	977	0.65	0.36	0.95	0.61	0.63	1.03	0.29
6	Myrcene	988	1.22	0.95	1.23	0.90	0.80	1.42	0.43
7	α-Terpinene	1016	0.04	-	0.05	0.02	0.04	-	-
8	*p*-Cymene	1023	0.07	0.03	0.07	0.05	-	-	-
9	Limonene	1027	0.44	0.25	0.45	0.32	0.31	0.45	0.19
10	1,8-Cineole	1032	5.16	3.65	7.78	5.70	8.19	8.77	4.76
11	(*E*)-β-Ocimene	1044	0.81	0.62	0.73	0.56	0.54	0.88	0.48
12	δ-Terpinene	1056	0.08	0.03	0.08	0.03	0.07	0.07	-
13	trans-Linalool oxide	1078	-	0.25	-	0.17	0.20	0.13	-
14	Terpinolene	1087	0.08	0.04	0.11	0.06	0.06	0.07	-
15	Linalool	1104	41.55	63.54	53.39	58.44	60.18	57.41	63.62
16	1-Octen-3-yl acetate	1110	0.15	0.12	0.12	0.14	0.11	0.10	0.11
17	3-Octanol acetate	1128	-	-	0.02	-	-	-	-
18	(*E*)-Myroxide	1140	0.17	0.26	0.33	0.29	0.22	0.26	0.19
19	Camphor	1146	0.72	0.85	1.06	0.62	0.61	0.73	0.51
20	Borneol	1167	0.20	0.17	0.26	0.33	0.32	0.23	0.19
21	Terpinene-4-ol	1177	0.40	0.15	0.37	0.15	0.39	0.21	0.21
22	α-Terpineol	1191	0.98	0.69	0.97	0.84	1.23	0.94	0.94
23	Methyl chavicol	1197	0.65	1.79	1.24	3.94	3.59	4.17	1.00
24	Octanol acetate	1209	0.64	0.42	0.53	0.36	0.48	0.29	0.31
25	Nerol	1230	-	0.07	-	0.04	-	-	-
26	Geraniol	1253	0.88	0.87	0.34	0.59	0.36	0.30	1.32
27	Bornyl acetate	1285	2.10	1.40	1.45	1.36	1.14	1.01	0.68
28	Myrtenyl acetate	1324	0.12	0.04	0.05	0.06	0.10	-	-
29	δ-Elemene	1341	0.11	0.06	0.11	0.07	0.16	-	-
30	α-Cubebene	1350	-	0.06	0.08	0.05	-	-	-
31	Eugenol	1359	6.53	2.05	2.53	2.10	3.13	2.82	2.26
32	Neryl acetate	1365	-	-	-	-	-	-	-
33	α-Copaene	1374	0.19	0.14	0.16	0.12	0.11	0.11	0.16
34	Geranyl acetate	1381	1.21	0.48	0.37	0.32	0.29	0.29	0.69
35	β-Elemene	1390	3.76	1.96	2.27	1.97	1.77	2.04	2.36
36	Methyl eugenol	1403	0.08	0.11	0.17	0.10	0.10	0.13	-
37	(*E*)-Caryophyllene	1418	0.65	0.30	0.56	0.40	0.35	0.23	0.43
38	trans-α-Bergamotene	1434	5.36	3.10	3.03	2.65	1.86	2.64	2.71
39	α-Guaiene	1437	0.92	0.44	0.49	0.40	0.36	0.45	0.52
40	α-Humulene	1453	1.51	0.55	0.72	0.61	0.56	0.58	0.77
41	allo-Aromadendrene	1461	0.39	0.21	0.31	0.26	0.19	0.21	0.29
42	Germacrene D	1480	3.26	1.84	2.30	1.76	1.58	1.79	2.81
43	β-Selinene	1489	-	-	0.10	0.13	-	-	-
44	Bicyclogermacrene	1495	0.88	0.48	0.51	0.47	0.44	0.44	0.62
45	α-Bulnesene	1504	1.97	1.09	1.18	0.95	0.83	1.01	1.17
46	δ-Cadinene	1513	2.84	1.69	1.99	1.77	1.31	1.49	1.96
47	trans-Calamenene	1521	0.65	0.35	0.38	0.39	0.27	0.32	0.41
48	(*E*)-Nerolidol	1561	0.26	0.13	0.19	0.14	0.10	-	-
49	Spathulenol	1577	0.82	0.64	0.70	0.58	0.37	0.39	0.43
50	Caryophyllene oxide	1583	0.28	0.17	0.28	0.23	0.15	-	-
51	1,10-di-epi-Cubenol	1614	1.13	0.82	0.95	0.84	0.73	0.55	0.71
52	epi-α-Cadinol	1642	7.16	5.06	5.87	5.47	4.21	4.20	5.29
53	β-Eudesmol	1650	0.26	0.04	-	0.04	-	-	-
54	α-Cadinol	1654	0.57	0.20	0.21	0.19	0.15	-	-
55	epi-α-Bisabolol	1687	-	-	0.36	0.22	-	-	-
	The number of compounds		47	49	51	52	46	40	35
	percent of known compounds		98.45	98.59	98.35	98.34	99.03	99.08	98.77

*: Refracting index; PSM: Psyllium seed mucilage.

**Table 2:** Essential oil components of treated basil by two industrial and plant HPs in various concentrations with application method in soil.

No.	Essential Oil Components	RI *	Control		Stockosorb^®^			PSM	
				0.1%	0.2%	0.3%	0.1%	0.2%	0.3%
1	α-Pinene	933	0.21	0.25	0.14	0.20	0.10	0.21	0.18
2	Camphene	948	0.07	0.07	-	0.08	0.04	0.09	0.06
3	Sabinene	972	0.27	0.23	0.12	0.17	0.12	0.24	0.20
4	β-Pinene	977	0.65	0.61	0.41	0.45	0.33	0.61	0.54
5	Myrcene	988	1.22	0.95	0.95	1.24	0.98	1.01	0.94
6	α-Terpinene	1016	0.04	-	-	0.03	-	0.02	0.02
7	*p*-Cymene	1023	0.07	-	-	0.04	-	0.03	0.03
8	Limonene	1027	0.44	0.34	0.25	0.28	0.22	0.31	0.28
9	1,8-Cineole	1032	5.16	5.10	3.28	3.83	3.62	5.09	5.10
10	(*E*)-β-Ocimene	1044	0.81	0.70	0.82	1.02	0.89	0.76	0.47
11	δ-Terpinene	1056	0.08	-	-	0.05	-	0.04	0.04
12	trans-Linalool oxide	1078	-	-	0.20	-	0.21	-	0.18
13	Terpinolene	1087	0.08	0.04	-	0.10	0.04	0.10	0.06
14	Linalool	1104	41.55	53.20	52.38	55.17	62.82	59.92	61.16
15	1-Octen-3-yl acetate	1110	0.15	0.09	-	0.09	0.09	0.08	0.09
16	(*E*)-Myroxide	1140	0.17	0.25	0.23	0.36	0.36	0.24	0.19
17	Camphor	1146	0.72	0.93	1.00	0.97	0.61	1.03	0.83
18	Borneol	1167	0.20	0.19	-	0.14	0.12	0.17	0.19
19	Terpinene-4-ol	1177	0.40	0.18	0.15	0.32	0.13	0.13	0.16
20	α-Terpineol	1191	0.98	0.89	0.64	0.69	0.57	0.67	0.74
21	Methyl chavicol	1197	0.65	1.89	-	2.89	0.49	0.94	0.19
22	Octanol acetate	1209	0.64	0.33	0.31	0.52	-	0.29	0.36
23	Nerol	1230	-	-	-	0.09	-	0.04	0.04
24	Geraniol	1253	0.88	1.02	3.07	1.36	0.22	0.95	0.97
25	Bornyl acetate	1285	2.10	1.13	0.78	1.14	1.09	0.77	0.79
26	Myrtenyl acetate	1324	0.12	-	-	-	-	0.06	0.04
27	δ-Elemene	1341	0.11	-	-	0.06	-	0.04	0.08
28	α-Cubebene	1350	-	-	-	0.05	-	-	0.07
29	Eugenol	1359	6.53	2.83	3.72	4.85	2.79	1.31	3.60
30	α-Copaene	1374	0.19	0.21	-	0.12	0.15	0.10	0.15
31	Geranyl acetate	1381	1.21	0.53	0.68	0.75	0.38	0.60	0.62
32	β-Elemene	1390	3.76	3.47	3.11	2.59	2.35	1.74	2.72
33	Methyl eugenol	1403	0.08	0.14	0.43	0.08	0.38	0.09	0.11
34	(*E*)-Caryophyllene	1418	0.65	0.58	0.59	0.39	0.32	0.36	0.29
35	trans-a-Bergamotene	1434	5.36	3.47	5.33	3.62	2.37	2.11	2.37
36	α-Guaiene	1437	0.92	0.81	0.74	0.59	0.59	0.35	0.60
37	α-Humulene	1453	1.51	0.96	1.40	0.71	0.78	0.47	0.72
38	allo-Aromadendrene	1461	0.39	0.37	0.28	0.25	0.26	0.16	0.24
39	Germacrene D	1480	3.26	3.06	2.67	2.04	2.07	1.50	2.41
40	β-Selinene	1489	-	-	0.48	0.15	-	-	-
41	Bicyclogermacrene	1495	0.88	0.93	1.00	0.62	0.60	0.46	0.70
42	α-Bulnesene	1504	1.97	1.80	1.66	1.37	1.37	0.83	1.37
43	δ-Cadinene	1513	2.84	2.44	2.07	1.73	2.06	1.25	1.61
44	trans-Calamenene	1521	0.65	0.49	0.49	0.38	0.37	0.25	0.33
45	(*E*)-Nerolidol	1561	0.26	0.17	0.22	0.14	0.16	0.11	0.19
46	Spathulenol	1577	0.82	0.50	0.89	0.60	0.87	0.43	0.64
47	Caryophyllene oxide	1583	0.28	-	0.28	0.23	0.22	0.11	0.17
48	1,10-di-epi-Cubenol	1614	1.13	0.93	0.97	0.78	0.98	0.58	0.76
49	epi-α-Cadinol	1642	7.16	6.64	6.31	4.98	6.23	3.76	4.86
50	β-Eudesmol	1650	0.26	-	-	-	-	-	0.05
51	α-Cadinol	1654	0.57	0.19	0.64	0.20	0.26	0.15	0.17
52	epi-α-Bisabolol	1686	-	-	-	0.17	-	-	-
	The number compounds		45	40	37	49	41	47	50
	percent of compounds		98.45	98.91	98.69	98.68	98.61	99.02	98.68

*: Refracting index; PSM: Psyllium seed mucilage.

**Table 3:** Soil nutrient analysis.

Texture	pH	Electrical Conductivity (Ds/ms)	OM%	OC%	*P*%	K %	N %
Sandy loam	7.8	1.21	0.64	0.37	0.002	0.023	0.064

OM%: Organic matter percentage, OC%: Organic carbon percentage.

**Table 4:** The amount of water used for each treatment during the 3 months growing period of basil.

		Water Used (L)			
	Control (0)	23			
		Stockosorb^®^		PSM	
		(Soil Application)	(Soil + Root Application)	(Soil Application)	(Soil + Root Application)
**Concentration**	0.1%	26	24	26	23
	0.2%	24	26	24	22
	0.3%	25	21	22	23

PSM: Psyllium seed mucilage.

## Supplementary Materials

The following are available online, Scheme S1: Plant phenylpropanoid pathway PAL, phenylalanine ammonia lyase; C4H, cinnamate 4-hydroxylase; 4CL, 4-coumaroyl-CoA ligase; CCR, cinnamoyl-CoA reductase; HCT, hydroxycinnamoyl-CoA shikimate/quinate hydroxycinnamoyl transferase; C3H, *p*-coumaroylshikimate 3’-hydroxylase; CAD, cinnamyl alcohol dehydrogenase; CAAT, coniferyl alcohol acetyl transferase; COMT, caffeic acid O-methyltransferase; CCOMT, caffeoyl-CoA.O-methyltransferase; F5H, ferulate-5-hydroxylase; CVS, chavicol synthase; EGS, eugenol synthase., Table S1: Information about the main constituents of the basil essential oil in limit allowed., Table S2: ANOVA for the traits evaluated in the manuscript.
