# Supplementary material for: Application of Super Absorbent Polymer and Plant Mucilage Improved Essential Oil Quantity and Quality of Ocimum basilicum var. Keshkeni Luvelou
Source: Molecules. 2020 May 28;25(11):2503. doi: 10.3390/molecules25112503 (PMC7321180; doi:10.3390/molecules25112503)
Supplement: Supplementary file 1 [file molecules-25-02503-s001.pdf]

molecules

Supplementary Materials

Application of Super Absorbent Polymer and Plant Mucilage Improved Essential Oil Quantity and Quality of *Ocimum basilicum* var. Keshkeni Luvelou

Somaye Beigi [1], Majid Azizi [2,*] and Marcello Iriti [3,*]

[1] Department of Horticultural Science, Faculty of Agriculture, Ferdowsi University of Mashhad, Mashhad 9177948974, Iran; sbeigi61@gmail.com
[2] Department of Horticultural Science, Faculty of Agriculture, Ferdowsi University of Mashhad, Mashhad 9177948974, Iran
[3] Department of Agricultural and Environmental Sciences, Milan State University, via G. Celoria 2, 20133 Milan, Italy
* Correspondence: azizi@um.ac.ir (M.A.); Tel.: +98-5138795618 (M.A.); marcello.iriti@unimi.it (M.I.); Tel.: +39-02-50316766 (M.I.)

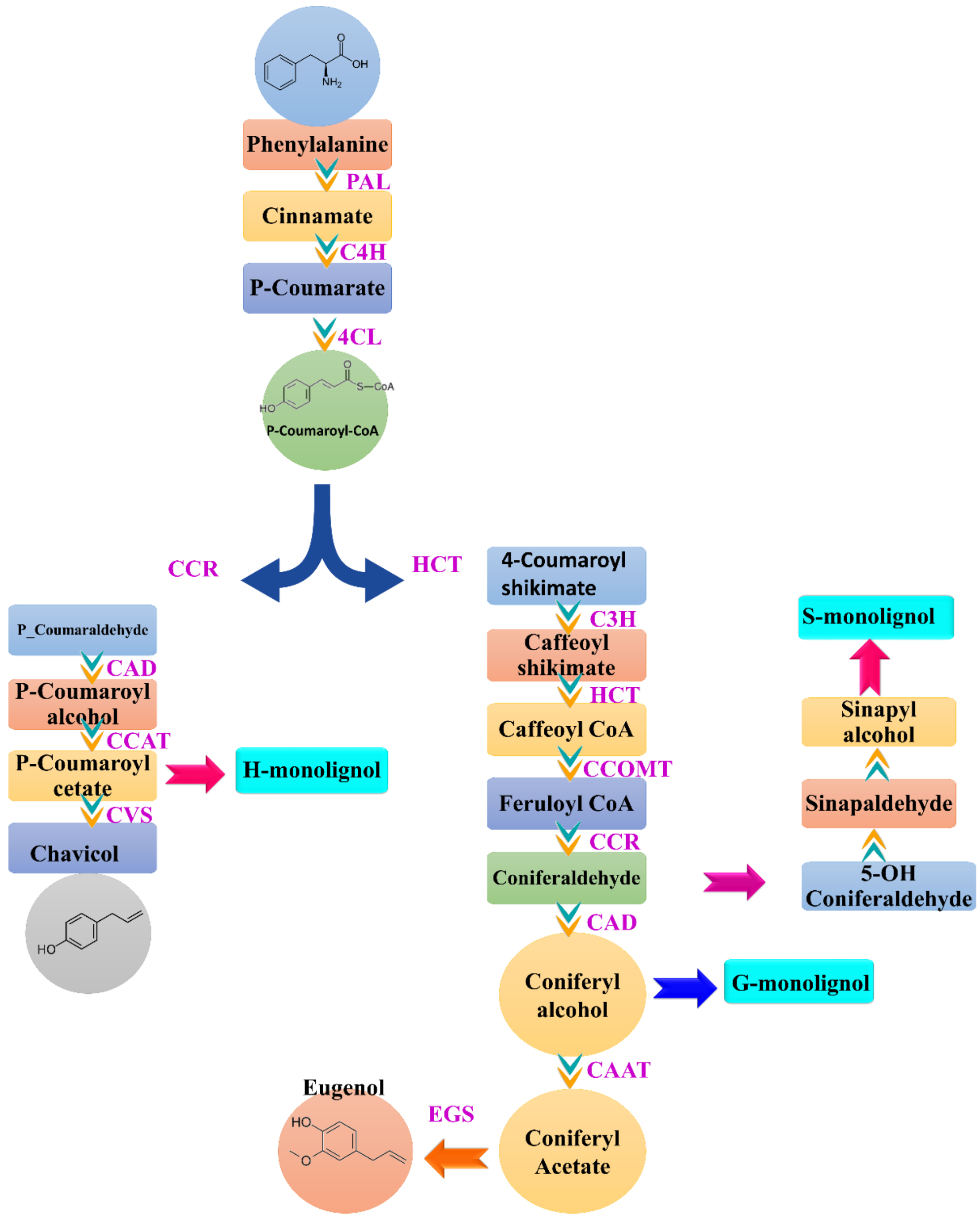

Scheme S1. Plant phenylpropanoid pathway PAL, phenylalanine ammonia lyase; C4H, cinnamate 4-hydroxylase; 4CL, 4-coumaroyl-CoA ligase; CCR, cinnamoyl-CoA reductase; HCT, hydroxycinnamoyl-CoA shikimate/quinate hydroxycinnamoyl transferase; C3H, *p*-coumaroylshikimate 3'-hydroxylase; CAD, cinnamyl alcohol dehydrogenase; CAAT, coniferyl alcohol acetyl transferase; COMT, caffeic acid *O*-methyltransferase; CCOMT, caffeoyl-CoA.O-methyltransferase; F5H, ferulate-5-hydroxylase; CVS, chavicol synthase; EGS, eugenol synthase.

Table S1. Information about the main constituents of the basil essential oil in limit allowed.

Main Constituent in Basil	Safety Level	Toxicity/Chemical of Concern	Medicinal/Food Properties	Toxic Effects Overdose	References
		20–50%			[1]
	<7.00 %<br>About 260mg per kg on average (about 23 grams [about 25mL] of basil essential oil for a 70kg adult)	Dose of 35 grams per day-about 47 grams (about 50mL) of Basil essential oil for a 70kg adult		Skin irritant	[2]
Methyl chavicol (estragole)		1–10 mg/kg body weight	Used in perfumes and as a food additive for flavor	Suspected to be carcinogenic and genotoxic. Psychoactive Effects	[3]
		Oral LD50 in rats: 1230 mg/kg<br>Oral LD50 in mice: 1250 mg/kg<br>Intraperitoneal LD50 in rats: 1030 mg/kg<br>Intraperitoneal LD50 in mice: 1260 mg/kg<br>Topical LD50 in rabbits: >5 gm/kg	Produces very mild sedative effects without any psychedelic effects		
Eucalyptol (1,8cineole)	0.002%	The acute oral LD50 is 2480 mg/kg (rat)	An ingredient in many brands of mouthwash and cough suppressant as well as an inactive ingredient in body powder<br>Used as an insecticide and insect repellent	Hazardous via ingestion, skin contact, or inhalation.<br>Acute health effects on behavior, Respiratory tract, and nervous system.<br>As a reproductive toxin for females and a suspected reproductive toxin for males.	[4]
		7.7–10%			[1,3]
		2%		Potential carcinogenic activity	[1]
Methyl eugenol	0.5% in fragrances<br>20 ppm in flavors<br>Oral-Rat: 1400.00 mg/kg	<6.00%			[5]

	Oral: LD50: 1.40 (g/kg)<br>Skin Rabbit: > 5000.00 mg/kg<br>Dermal: LD50: >5.00				
		1%			[1]
Camphor	Doses in adults are in the range 50–500 milligrams per kilogram (mg/kg) of body weight.	2 grams (g) causes serious toxicity and 4 grams is potentially lethal	Readily absorbed through the skin and produces a cool feeling<br>Acts as a slight local anesthetic and antimicrobial<br>Effective as a cough suppressant<br>An active ingredient (along with menthol) in vapor-steam products<br>Administered orally in small quantities (50 mg) for minor heart symptoms and fatigue<br>Used for the skin	Poisonous when ingested and can cause seizures, confusion, irritability<br>Neuromuscular hyperactivity	[6]
		< 4.00 %		Sensitizer (skin Irritant)	[5]
Eugenol			Used in perfumes, flavorings<br>Essential oils and in medicine as a local antiseptic and anesthetic which has restorative and prosthodontic applications in dentistry	Hepatotoxic, causing a wide range of symptoms from blood in the patient's urine<br>Convulsions<br>Diarrhea<br>-Nausea<br>-Unconsciousness<br>-Dizziness<br>-Rapid heartbeat	[7]
Safrole				Carcinogenic (cancer causing–especially liver tumors)	[2]
α- and β-thujones	0.5mg/kg	<10 mg/L	Immune-system stimulation effects	Cause convulsions associated with lesions of the cerebral cortex Anxiety<br>Sleeplessness<br>Toxic to brain, kidney and liver cells and could cause convulsions	[8]

Table S2. ANOVA for the traits evaluated in the manuscript.

S.O.V	d.f	Essential Oil Yield	Essential Oil Content	WUE	Dry Matter
Replication	2	0.0004^{ns}	0.0023^{ns}	0.0011^{ns}	0.0087^{ns}
Application method	1	0.0004^{**}	0.0038^{**}	2.4075^{**}	27.999^{**}
HPs type	1	0.0007^{ns}	0.0063^{**}	0.0841^{**}	0.8586^{**}
HPs concentration	3	0.0159^{**}	0.1100^{**}	1.7284^{**}	58.219^{**}
Application method× HPs concentration	3	0.0043^{**}	0.0048^{**}	0.4884^{**}	6.3073^{**}
HPs type× HPs concentration	3	0.0040^{**}	0.0132^{**}	0.3089^{**}	6.0609^{**}
Application method× HPs type	1	0.0011^{ns}	0.1530^{**}	0.9947^{**}	27.968^{**}
Application method× HPs type× HPs concentration	3	0.0080^{**}	0.0237^{**}	0.4195^{**}	5.8888^{**}
Error	30	0.002	0.0137	0.2645	6.1046

S.O.V: Sources of Variation; d.f: Degree of Freedom; HPs: Hydrophilic Polymers. ns and **: None significant and significant at 0.01 probability level respectively.

References

1. Silano, V.; Speijers, G.; Müller, U.B.S.; Dusemund, B.; Loulergue, M.H.; Lugasi, A.; Toth, J. EFSA Compendium of botanicals that have been reported to contain toxic, addictive, psychotropic or other substances of concern. *EFSA J.* **2009**, *7*, 281.
2. Gupta, Suresh, K. "Ocimum sanctum modulates selenite-induced cataractogenic changes and prevents rat lens opacification." *Curr. Eye Res.* **2005**, *30*, 583–591.
3. Speijers, G.; Bottex, B.; Dusemund, B.; Lugasi, A.; Tóth, J.; Amberg-Müller, J.; Rietjens, I.M. Safety assessment of botanicals and botanical preparations used as ingredients in food supplements: Testing an European Food Safety Authority-tiered approach. *Mol. Nutr. Food Res.* **2010**, *54*, 175–185.
4. Carratù, B.; Federici, E.; Gallo, F.R.; Geraci, A.; Guidotti, M.; Multari, G.; Sanzini, E. Plants and parts of plants used in food supplements: An approach to their safety assessment. *Ann. Dell'istituto Super. Di Sanita* **2010**, *46*, 370–388.
5. Dweck, A.C. Toxicology of essential oils reviewed. Person. *Care Mag.* **2009**, *2*, 65–72.
6. Chen, W.; Vermaak, I.; Viljoen, A. (2013). Camphor—A fumigant during the black death and a coveted fragrant wood in ancient Egypt and Babylon—A review. *Molecules* **2003**, *18*, 5434–5454.
7. Giuliani, F. The Composition, Structure, Sources, and Applications of Eugenol. *ESSAI* **2014**, *12*, 19.
8. Aqil, M.; Ahad, A.; Sultana, Y.; Ali, A. Status of terpenes as skin penetration enhancers. *Drug Discov. Today* **2007**, *12*, 1061–1067.

Sample Availability: Samples of the compounds are not available from the authors.

© 2020 by the authors. Licensee MDPI, Basel, Switzerland. This article is an open access article distributed under the terms and conditions of the Creative Commons Attribution (CC BY) license (http://creativecommons.org/licenses/by/4.0/).
